# Single-cell RNA-seq reveals cellular heterogeneity of mouse carotid artery under disturbed flow

**DOI:** 10.1038/s41420-021-00567-0

**Published:** 2021-07-16

**Authors:** Fengchan Li, Kunmin Yan, Lili Wu, Zhong Zheng, Yun Du, Ziting Liu, Luyao Zhao, Wei Li, Yulan Sheng, Lijie Ren, Chaojun Tang, Li Zhu

**Affiliations:** 1 Cyrus Tang Hematology Center, Suzhou, Jiangsu China; 2 Collaborative Innovation Center of Hematology, Suzhou, Jiangsu China; 3 Suzhou Key Laboratory of Thrombosis and Vascular Diseases, Suzhou, Jiangsu China; 4 National Clinical Research Center for Hematologic Diseases, The First Affiliated Hospital of Soochow University, Suzhou, Jiangsu China; 5 State Key Laboratory of Radiation Medicine and Protection Soochow University, Suzhou, Jiangsu China

**Keywords:** Cell biology, Biotechnology

## Abstract

Disturbed blood flow (d-flow) has been known to induce changes of the cells in the arterial wall, increasing the risk of atherosclerosis. However, the heterogeneity of the vascular cell populations under d-flow remains less understood. To generate d-flow in vivo, partial carotid artery ligation (PCL) was performed. Seven days after ligation, single-cell RNA sequencing of nine left carotid arteries (LCA) from the PCL group (10,262 cells) or control group (14,580 cells) was applied and a single-cell atlas of gene expression was constructed. The integrated analysis identified 15 distinct carotid cell clusters, including 10 d-flow-relevant subpopulations. Among endothelial cells, at least four subpopulations were identified, including Klk8^hi^ ECs, Lrp1^hi^ ECs, Dkk2^hi^ ECs, and Cd36^hi^ ECs. Analysis of GSVA and single-cell trajectories indicated that the previously undescribed Dkk2^hi^ ECs subpopulation was mechanosensitive and potentially transformed from Klk8^hi^ ECs under d-flow. D-flow-induced Spp1^hi^ VSMCs subpopulation that appeared to be endowed with osteoblast differentiation, suggesting a role in arterial stiffness. Among the infiltrating cell subpopulations, Trem2^hi^ Mφ, Birc5^hi^ Mφ, DCs, CD4^+^ T cells, CXCR6^+^ T cells, NK cells, and granulocytes were identified under d-flow. Of note, the novel Birc5^hi^ Mφ was identified as a potential contributor to the accumulation of macrophages in atherosclerosis. Finally, Dkk2^hi^ ECs, and Cd36^hi^ ECs were also found in the proatherosclerotic area of the aorta where the d-flow occurs. In conclusion, we presented a comprehensive single-cell atlas of all cells in the carotid artery under d-flow, identified previously unrecognized cell subpopulations and their gene expression signatures, and suggested their specialized functions.

## Introduction

Disturbed flow (d-flow) is characterized by low and oscillatory shear stress, which plays a vital role in the development of atherosclerosis. In regions of d-flow, the blood vessels and the cells of the arterial wall undergo significant changes, including endothelial dysfunction, inflammation, angiogenesis, EndMT, apoptosis, extracellular matrix (ECM) remodeling, VSMCs (vascular smooth muscle cells) migration, and arterial stiffening [1–8]. These changes increase the risk of atherosclerosis in the vascular wall [9–11]. Using microarray analysis, previous studies have reported numerous shear-sensitive genes in mouse and human endothelial cells (ECs) and identified the critical roles they play in various biological processes [7, 12–16]. For instance, using cultured human ECs, early studies identified some shear-sensitive genes, such as zinc-finger protein EZF/GKLF [12], Kruppel-like factor 2 (Klf2) [13], intercellular adhesion molecule 1 (ICAM-1) [14], bone morphogenic protein 4 (BMP4) [15], and Angiopoietin-2 (Ang2) [16]. Subsequent studies by collecting endothelial-enriched RNAs from the carotid artery identified numerous mechanosensitive genes, including LIM domain only 4 (Lmo4) [17], DNA methyltransferase-1, and miR-712 [18]. In addition, the d-flow may also induce phenotypic changes in other vascular cells indirectly or even directly [19]. Although the d-flow induces the expression of mechanosensitive genes and activation of certain cellular pathways in the major arterial cell types of the vasculature, the molecular profile, and the heterogeneity of individual cells are poorly understood.

Single-cell RNA sequencing (scRNA-seq) provided a detailed analysis of cell populations at the single-cell level [20]. Several scRNA-seq studies characterized EC heterogeneity in single tissues [21–23] and multiple tissues [24, 25]. Of note, Vcam1^+^ ECs and Cd36^+^ ECs in the aorta exhibited phenotypic heterogeneity [26]. Since the Vcam1^+^ subpopulation is spatially located in the lesser curvature of the aorta, a region of disturbed blood flow commonly occurs, it was proposed that the unique distribution patterns for Vcam1^+^ ECs and Cd36^+^ ECs subtypes in the lesser and greater curvatures of the aorta may be the result of differences in blood flow and shear stress in these regions [26]. Studies using scRNA-seq on the infiltration of immune cells in the atherosclerotic aorta showed that aortic CD45^+^ leukocytes are heterogeneous and have a particular set of functions, such as lipid metabolism and inflammation [27, 28]. Two groups of VSMCs (SMC1 and SMC2) were identified in the aorta and notably, the modulated SMCs were transformed into unique fibroblast-like cells in the development of atherosclerosis [29].

Although significant progresses in understanding the role of d-flow in endothelial cells have been obtained using in vitro approaches [12, 14], those methods are unlikely to provide the in vivo microenvironment and often have their limitations for the translational purpose. To obtain the direct in vivo evidence that the d-flow induces the heterogeneity of blood vessel cell subtypes, we performed PCL surgery, prepared the d-flow-stimulated vascular cells followed by scRNA-seq, and constructed a single-cell atlas of gene expression. We identified 10 distinct d-flow-associated cell subpopulations, involving ECs, VSMCs, macrophages, dendritic cells, lymphocytes, and granulocytes. Compared to the unstimulated carotid vascular cells, these clusters presented their unique gene signatures and displayed the enrichment of specific functions, such as angiogenesis, leukocyte chemotaxis, complement activation, immune response, cell killing, leukocyte aggregation, and osteoblast differentiation. Importantly, Dkk2^hi^ ECs and Cd36^hi^ ECs were found in the proathrosclerotic area of the aorta where the d-flow occurs, indicating their potential involvement in atherosclerosis.

## Results

### Single-cell profile of d-flow-associated cell populations in carotid arteries

**Fig. 1:**
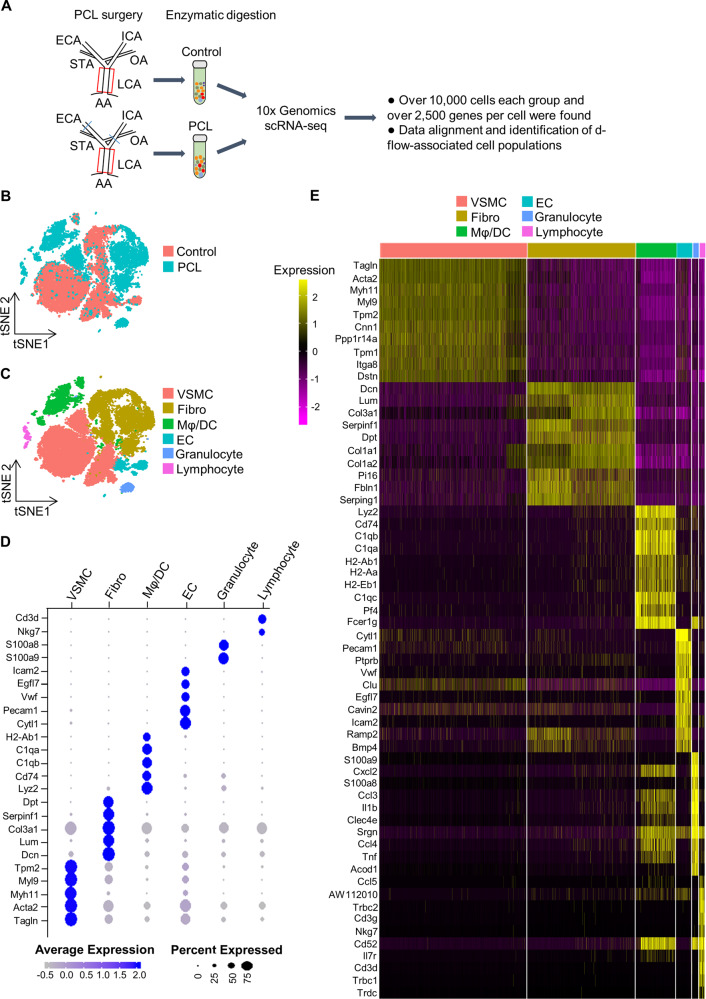
Single-cell RNA-sequencing (scRNA-seq) atlas of left carotid artery cell types. **A** Schematic overview of experimental design. **B** t-distributed stochastic neighbor embedding (t-SNE) represents the aligned gene expression data in single cells extracted from the left carotid artery of wild-type mice 7 days after partial carotid ligation (PCL) surgery or without PCL (control). **C** t-SNE representation of single-cell gene expression shows the identified major left carotid artery cell types. **D** The top markers defining each type of cell cluster in **C** are listed. The size of each circle represents the proportion of cells within the group expressing each transcript. The blue color dots indicated highly expressed genes, while gray color dots correspond to low expressed genes. **E** The heatmap showing the 10 most upregulated genes in each cluster defined. ECA external carotid artery, ICA internal carotid artery, OA: occipital artery, STA superior thyroid artery, LCA left carotid artery, AA aortic arch, EC endothelial cells, VSMC vascular smooth muscle cells, fibro fibroblasts, Mφ/DC macrophages and dendritic cells.

To reveal the changes in blood vessels under disturbed blood flow, we performed scRNA-seq for the left carotid arteries (LCA) with or without partial carotid ligation (PCL) surgery by using a 10x Genomics platform (Fig. 1A). Nine arteries in each group were sequenced and >2500 genes per cell were found after enzymatic dissociation with collagenase/deoxyribonuclease/trypsin (Fig. S2A, B). After removing the low-quality cells, 10,262 cells from the PCL group and 14,580 cells from the control (non-PCL) were used for integrated single-cell RNA-seq analysis. D-flow-associated cell populations and the control cells were visualized using t-stochastic neighbor embedding (t-SNE) (Fig. 1B).

Using 2000 variable genes with similar profiles, unsupervised Seurat-based clustering showed the cell types of both the PCL and control groups. Mainly six cell types, including VSMCs, fibroblasts, ECs, and d-flow-induced immune cell populations (Mφ/DCs, granulocytes, and lymphocytes) from the carotid arteries were identified by known canonical markers (Fig. 1C, Figure S3A, B). The marker genes that had been reported in the aorta [26, 29] have high-quality specificity in the carotid arteries. For example, the fibroblast-specific marker genes (Dpt, Serpinf1, Col3a1, Lum, Dcn) in the aortic fibroblasts were highly expressed in the cell cluster in the carotid arteries (Fig. 1D).

To further analyze the specificity of cell types, we identified all genes of each cell type with log-fold enrichment >2 relative to all other cells. The heatmap showed the top 10 differentially expressed genes of each cell type (Fig. 1E). Notably, we found that Cavin2 and Ppp1r14a may serve as novel marker genes besides genes that had been reported in mouse aorta [26, 29]. Moreover, Cavin2 (caveolae associated 2), a critical gene in the maintenance and function of endothelial cells [30], was highly expressed in the EC cluster, while Ppp1r14a (also known as Cpi17), a gene that plays a pivotal role in VSMC differentiation [31], had expression specificity in the VSMC cluster. Therefore, Cavin2 and Cpi17 are potential molecular markers to identify the endothelial cells and vascular smooth muscle cells in mice, respectively. Together, our results of single-cell RNA sequencing using the 10x Genomics platform identified cell populations of the carotid artery under disturbed blood flow.

### Functional heterogeneity of EC subpopulations under d-flow

**Fig. 2:**
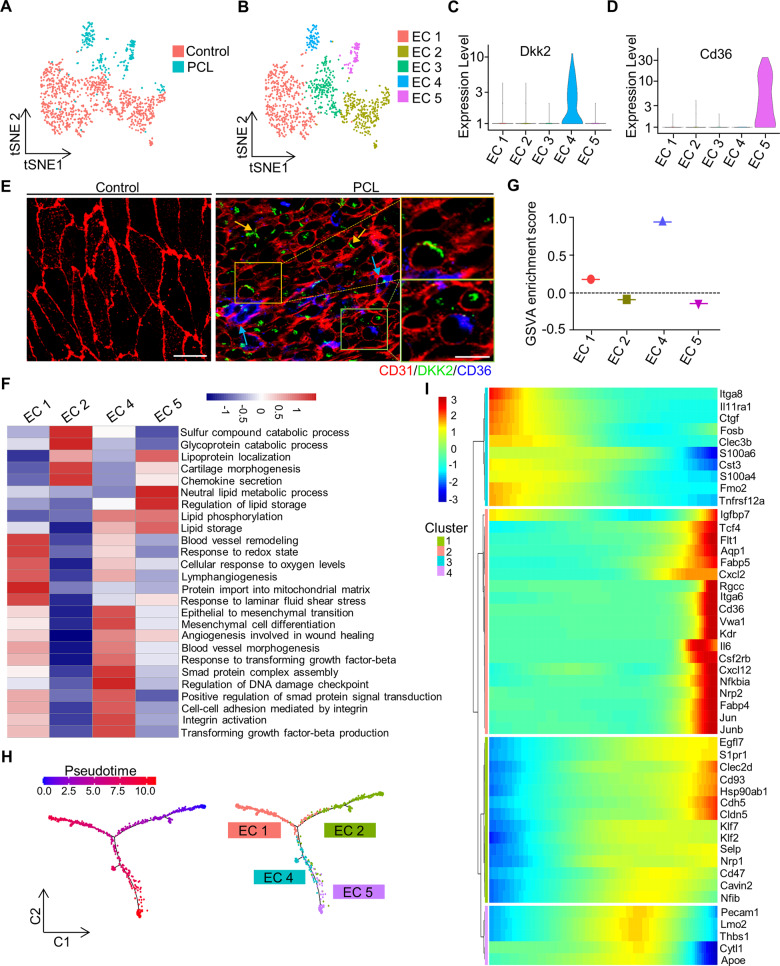
Identification of endothelial cell clusters under disturbed blood flow. **A** t-distributed stochastic neighbor embedding (t-SNE) plot of EC subpopulations from the left carotid artery of wild-type mice 7 days after partial carotid ligation (PCL) or without PCL (control). **B** t-SNE plot of five distinct EC clusters from the PCL and control. **C** The violin plots of Dkk2 (an EC 4-associated marker) expression in all five EC clusters. **D** The violin plots of Cd36 (an EC 5-associated marker) expression in all five EC clusters. **E** Representative en face immunostaining images for CD36 and DKK2 in the left carotid artery with partial carotid ligation (right) or without PCL (control) (left) (red: CD31; green: DKK2; blue: CD36). bar = 20 µm. The magnified image was shown on the right, where the arrows indicated the co-localization of the related proteins. bar = 10 µm. **F** The heatmap showing the differences in biological processes by GSVA enrichment scores among the different EC clusters. **G** GSVA enrichment scores by using upregulated gene set (Fosl2, Ctgf, Ctps, Lmo4, Angpt2, Icam1, Bmp4, and Sema7a) under d-flow. **H** Branched pseudotime trajectory, each cell being colored by its pseudotime value (left) and its seurat clusters (right). **I** The heatmap showing variable genes along the pseudotime trajectory. The *X-*axis represents cells ordered by the pseudotime (from left to right) and different colors correspond to the scaled expression of each gene in each cell (from Monocle).

To observe heterogeneity of ECs in blood vessels when the d-flow occurs, we examined 1172 endothelial cells from the PCL and control groups (Fig. 2A). Unsupervised Seurat-based clustering of 1172 endothelial cells revealed five distinct subpopulations (Fig. 2B). EC 1 (475 cells) and EC 2 (223 cells) were dominantly found from the control group, whereas EC 4 (82 cells) and EC 5 (78 cells) are d-flow-derived EC subpopulations. EC 3 (314 cells) represented endothelial cells that presented in both the d-flow stimulated and the control groups, which was disregarded in subsequent analysis. Of note, the Klk8 gene expression in EC 1 was higher than other clusters (*P* < 1.1×10^−^^78^ by Wilcoxon rank-sum test), while Lrp1 (low-density lipoprotein receptor-related protein 1) was highly expressed in EC 2 (*P* < 5.9×10^−^^40^ by Wilcoxon rank-sum test) (Figure S4A). Notably, the most significantly enriched gene of EC 4 by d-flow was Dkk2 (dickkopf WNT signaling pathway inhibitor 2) (Fig. 2C), which was previously reported to play an essential role in controlling angiogenesis [32]. Likewise, the most significantly enriched gene of EC 5 was Cd36 (Fig. 2D), which encodes a receptor for oxidized low-density lipoprotein gene for lipid metabolism [33]. Thus, these clusters can be referred to as Klk8^hi^ ECs, Lrp1^hi^ ECs, Dkk2^hi^ ECs, and Cd36^hi^ ECs.

To identify the spatial location of d-flow-related EC subpopulations, we chose Dkk2 and Cd36 as the markers of Dkk2^hi^ ECs and Cd36^hi^ ECs subpopulations, respectively. En face immunostaining showed the expression of CD36 and DKK2 in the left common carotid artery after PCL surgery, but not in the non-PCL controls (Fig. 2E, Figure S4B), indicating that Dkk2^hi^ ECs and Cd36^hi^ ECs are the two subpopulations of endothelial cells induced by d-flow.

In order to identify the potential functions of each EC subpopulation, gene set variation analysis (GSVA) was used to analyze each cell subpopulation. Analysis of biological process gene sets from the Molecular Signatures Database highlighted the functional heterogeneity among the EC subpopulations (Fig. 2F). Compared to normal EC clusters (EC 1 and EC 2), The lipid phosphorylation and lipid storage scores were higher in d-flow-derived EC subpopulations (EC 4 and EC 5). Further analysis of endothelial cells from these four clusters showed that EC 4 had other high-scored and d-flow-related biological processes, such as epithelial to mesenchymal transition [34], integrin activation [35], and transforming growth factor-beta production [36]. Since these biological functions are manifested under d-flow, we proposed that EC 4 (Dkk2^hi^ ECs) is a mechanosensitive EC subpopulation. To test this hypothesis, we performed GSVA by designing a gene set in which genes are upregulated when ECs undergo disturbed flow, including Lmo4, Angpt2, Icam1, Ctgf, Ctps, Sema7a, Fosl2, and Bmp4. Surprisingly, we found that the enrichment score of the EC 4 cluster was relatively higher than others (Fig. 2G, Figure S4C). On the contrary, the antiatherosclerosis genes that are downregulated by d-flow, such as Klf2, Klk10, and Nos3, were weakly expressed in EC 4 (Figure S4D). These results indicated that Dkk2^hi^ ECs cluster (EC 4) was a d-flow-sensitive EC subpopulation.

To investigate the transition of the EC clusters, we applied single-cell trajectories analysis and showed that the cells formed a continuous progression starting from EC 2 cluster, then Klk8^hi^ ECs and Dkk2^hi^ ECs, and progressively ending toward EC 5 cluster (Fig. 2H). In this trajectory, the starting cells expressed high levels of markers, such as Clec3b, S100a4, and Fmo2, while the strongly expressed genes were enriched at the end of cell transition, which includes chemokines and cytokines (e.g., Cxcl2, Cxcl12, and Il6), and transcription factors (e.g., Klf2, Klf7, Jun, and Tcf4) (Fig. 2I). These results indicated that the Dkk2^hi^ EC cluster was the first group of transition cells in the d-flow-derived EC clusters and that Klk8^hi^ ECs, which respond to laminar fluid shear stress (Fig. 2F) might directly transform into Dkk2^hi^ ECs under d-flow.

### Novel VSMCs subpopulation under d-flow

**Fig. 3:**
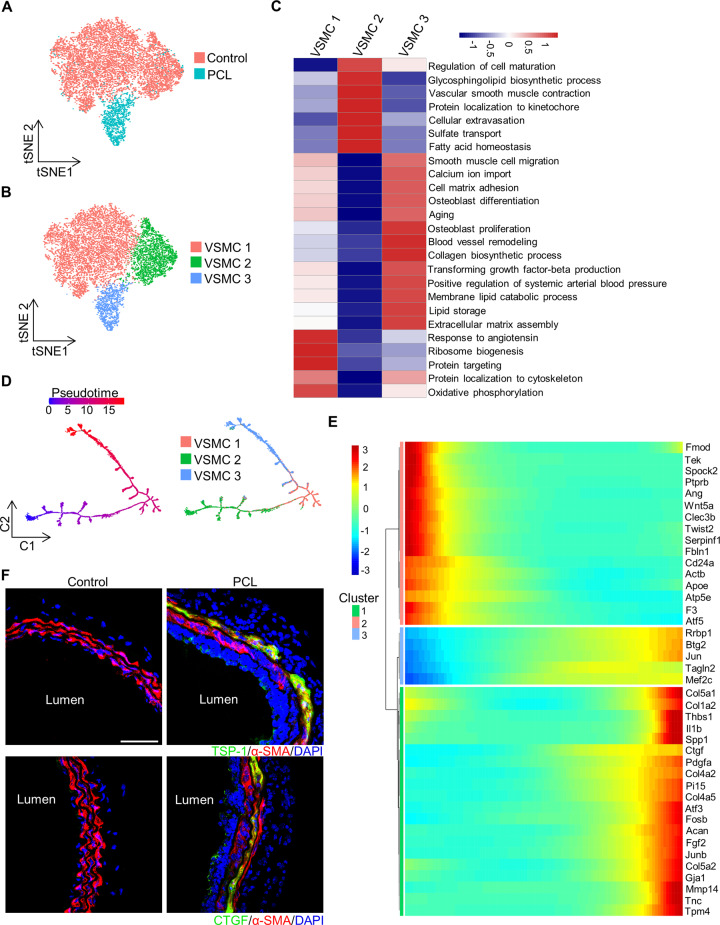
Identification of vascular smooth muscle cell clusters under disturbed blood flow. **A** t-distributed stochastic neighbor embedding (t-SNE) representation of aligned gene expression data in each individual cell extracted from the left carotid artery of wild-type mice 7 days after partial carotid ligation (PCL) or without PCL (control). **B** t-SNE plot of three VSMCs clusters from the PCL and control. **C** The heatmap showing differences of biological processes among three distinct VSMCs by GSVA. **D** Branched pseudotime trajectory, each cell being colored by its pseudotime value (left) and its seurat clusters (right). **E** The heatmap showing variable genes along the pseudotime trajectory. The *X-*axis represents cells ordered by pseudotime (from left to right) and different colors correspond to the scaled expression of each gene in each cell (from Monocle). **F** Representative immunofluorescence microscopy images for TSP-1 and CTGF in the carotid artery from wild-type mice with partial carotid ligation (PCL) or control. bar = 50 µm.

VSMCs (11,383 cells) from the PCL and control group were examined (Fig. 3A). Unsupervised Seurat-based clustering showed that these VSMCs can be divided into three distinct clusters, including VSMC 1, VSMC 2, and VSMC 3 (Fig. 3B). Further analysis of these clusters showed that the VSMC 3 (1387 cells) was a d-flow-associated subpopulation, while VSMC 1 (7332 cells) and VSMC 2 (2664 cells) were two clusters of normal VSMCs from the control group. Of note, Spp1 (secreted phosphoprotein 1) was highly expressed in VSMC 3 (*P* = 0 by Wilcoxon rank-sum test), which is an osteoblastic marker. Thus, VSMC 3 can be referred to as Spp1^hi^ VSMCs. To investigate the functional heterogeneity of these VSMCs subpopulations, we scored the biological process in each cluster by GSVA (Fig. 3C). Compared to normal VSMCs, Spp1^hi^ VSMCs had a particular set of functions such as osteoblast differentiation, collagen biosynthetic process, blood vessel remodeling, aging, and positive regulation of systemic arterial blood pressure. Notably, these biological processes are essential mediators of arterial stiffness according to previous reports [37–40].

We applied pseudotime analysis to investigate the gene profile changes of these three VSMCs subpopulations. The group labels showed that the VSMCs formed a continuous progression that started with VSMC 2, then VSMC 1, and finally Spp1^hi^ VSMCs (Fig. 3D). The cells at the beginning of this trajectory expressed high levels of markers, such as Twist2, Clec3b, and Actb, while the end of the trajectory was enriched in cells expressing osteoblast differentiation-associated genes (e.g., Mef2c, Tnc, Gja1, Junb, and Tpm4) and profibrotic genes (e.g., Ctgf, Col5a2, and Col5a1) (Fig. 3E). Importantly, Thbs1 (thrombospondin 1), a d-flow-related gene that promotes arterial stiffening [7], was highly upregulated at the end of the trajectory. Of note, since most of the cells in the Spp1^hi^ VSMCs group were at the end of this trajectory, we realized d-flow promoted the expression of these genes. Consistent with the pseudotime analysis, immunofluorescence staining showed the upregulated expression of profibrotic TSP-1 and CTGF after PCL (Fig. 3F). Together, our results indicated that the Spp1^hi^ VSMCs may play a role in arterial stiffness induced by d-flow.

### Distinct gene expression profiles of four infiltrated Mφ/DCs subpopulations

**Fig. 4:**
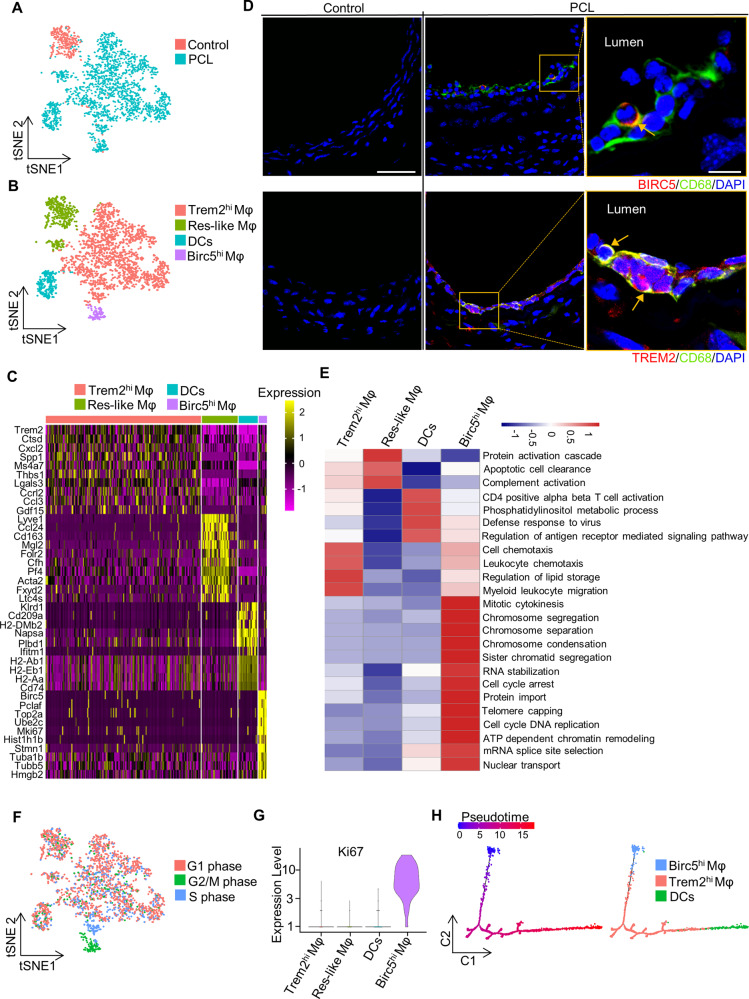
Distinct macrophage populations and their expression signatures. **A** t-distributed stochastic neighbor embedding (t-SNE) plot of macrophages (Mφ)-dendritic cells (DCs) populations from the left carotid artery of wild-type mice 7 days after partial carotid ligation (PCL) or without PCL (control). **B** t-SNE plot of four Mφ-DCs clusters from the PCL and control. **C** The heatmap showing 10 most upregulated genes in each defined cluster. **D** Representative immunofluorescence microscopy images for BIRC5 and TREM2 in the left carotid artery with partial carotid ligation. The left panel is the control group, bar = 50 µm. The middle panel is the merged images either stained with CD68 (green) + BIRC5 (red) (top) or CD68 (green) + TREM2 (red) (bottom). The right panel is the enlarged image of the box in the middle panel. The arrow represents the co-localization of the related proteins. bar = 10 µm. **E** The heatmap of GSVA enrichment scores in biological processes among four distinct populations. **F** Cell-cycle analysis. Mφ/DCs were evaluated by calculating cell-cycle phase scores based on the canonical markers. **G** The violin plot of Ki67 expression in all four clusters. **H** Branched pseudotime trajectory, each cell population being colored by its pseudotime value (left) and its seurat clusters (right).

We examined 2536 macrophages and dendritic cells from the PCL and control groups (Fig. 4A). These cells clustered into four distinct groups, including Trem2^hi^ Mφ, Res-like Mφ, DCs, and Birc5^hi^ Mφ (Fig. 4B). We found that Res-like Mφ was mainly from the control group, while Trem2^hi^ Mφ, DCs, and Birc5^hi^ Mφ were dominantly from the PCL group, implying that the d-flow promotes the presence of different macrophages and DCs in the carotid artery. The heatmap showed the expression of the top 10 significantly enriched genes in each cluster (Fig. 4C). Immunofluorescence staining showed the infiltration of Birc5^hi^ Mφ (Fig. 4D, upper panel) or Trem2^hi^ Mφ after PCL (Fig. 4D, lower panel, as indicated by arrows). Consistently, Trem2^hi^ Mφ was previously reported in mouse atherosclerotic aorta [27].

To investigate the functional heterogeneity of macrophages and dendritic cells, we scored the biological processes in each cluster by GSVA (Fig. 4E). Trem2^hi^ Mφ had a particular set of functions, such as cell chemotaxis, myeloid leukocyte migration, and regulation of lipid storage, which is consistent with the previous report [27]. DCs had the functions of defense response to virus and antigen presentation, while Res-like Mφ plays a vital role in clearing apoptotic cells and complement activation. Surprisingly, we noticed that Birc5^hi^ Mφ showed distinctive functions related to proliferation, such as chromosome segregation, RNA stabilization, cell-cycle DNA replication, and mRNA splice site selection (Fig. 4E, Figure S5A). Moreover, the cell-cycle analysis revealed that most of Birc5^hi^ Mφ were in the G2/M phase (Fig. 4F). Of note, Ki67, a known proliferation marker expressed throughout the cell cycle had a specific high expression in Birc5^hi^ Mφ (Fig. 4G). Together, these data suggested that the Birc5^hi^ Mφ exhibit a high proliferation ability.

We next assessed the role of Birc5^hi^ Mφ in d-flow-induced Mφ/DCs. Single-cell trajectories analysis showed that Mφ/DCs formed a continuous progression starting in Birc5^hi^ Mφ and progressively ending toward DCs (Fig. 4H), indicating that Birc5^hi^ Mφ may be proliferated rapidly and potentially transformed into DCs in response to d-flow.

### D-flow-induced presence of lymphocytes and granulocytes in the vessel wall

**Fig. 5:**
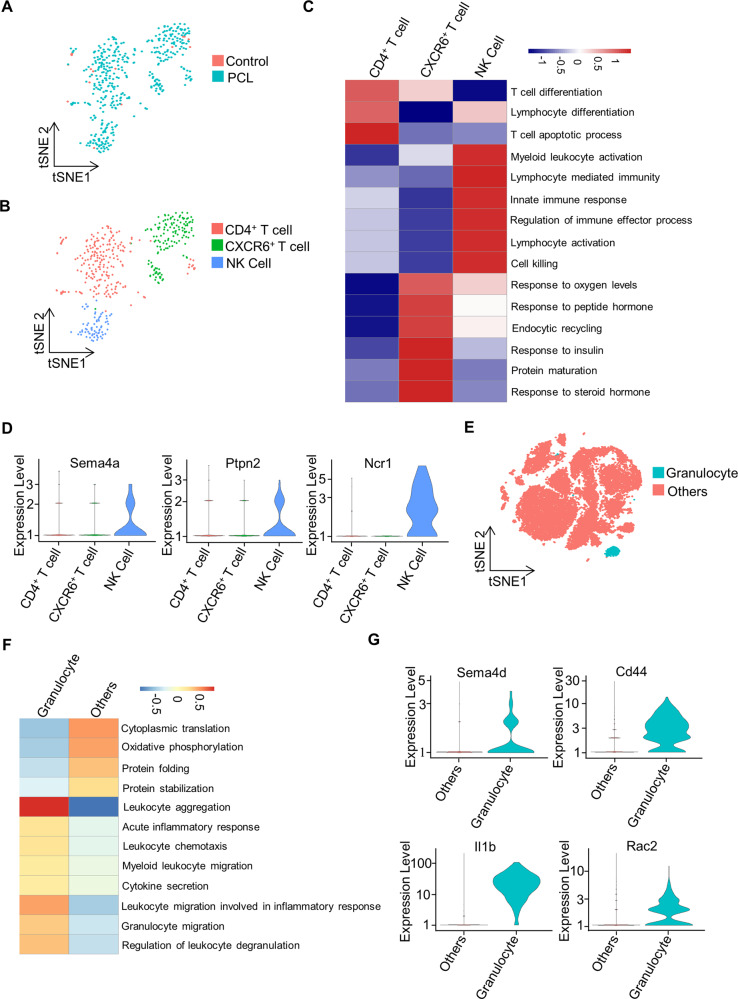
The infiltration of lymphocytes and granulocytes in carotid artery. **A** T-Distributed Stochastic Neighbor Embedding (t-SNE) plot of lymphocyte subpopulations from the left carotid artery of wild-type mice 7 days after partial carotid ligation (PCL) or without PCL (control). **B** t-SNE plot of three lymphocyte clusters from LCA with PCL or control, including CD4^+^ T cells, CXCR6^+^ T cells, and NK cells. **C** The heatmap showing differences of biological processes among three distinct lymphocyte populations by GSVA. **D** The violin plots of the expression of Sema4a, Ptpn2, or Ncr1 in CD4^+^ T cells, CXCR6^+^ T cells, or NK cells. **E** t-SNE plot of populations of granulocyte and others. **F** The heatmap showing the biological processes of granulocyte population compared to others (without scaled). **G** The violin plots represent the expression of Sema4d, Cd44, Il1b, or Rac2 in granulocyte.

We examined the presence of lymphocytes (442 cells) in the carotid artery when the d-flow occurs (Fig. 5A) and found three clusters of lymphocytes, including CD4^+^ T cells (enriched genes: Cd4, Tcf7, and Trbc2), CXCR6^+^ T cells (Cxcr6, Il7r, and Cd3g), and NK cells (Gzma, Ccl5, and Klrb1c) (Fig. 5B). We then calculated GSVA enrichment scores among these clusters and noticed that CD4^+^ T cells were involved in T cell differentiation and apoptosis, while CXCR6^+^ T cells responded to oxygen levels, insulin, and hormone, and participated in endocytic recycling and protein maturation. The NK cells were shown to be involved in the activation of lymphocytes and myeloid leukocytes, lymphocyte-mediated immunity, innate immune response, regulation of immune effector process, and cell killing (Fig. 5C). Furthermore, genes regulating lymphocyte activation, such as Sema4a, Ptpn2, and Ncr1 were highly expressed in NK cells, compared with other clusters (Fig. 5D).

We also computed GSVA enrichment scores of granulocytes (468 cells) (Fig. 5E). The heatmap showed that granulocytes were involved in various biological processes, such as leukocyte aggregation, acute inflammatory response, granulocyte migration, and especially leukocyte aggregation (Fig. 5F). Moreover, we found that Sema4d, Cd44, Il1b, and Rac2, genes that encode molecules involved in leukocyte aggregation, were upregulated in granulocytes (Fig. 5G).

### Presence of Dkk2^hi^ ECs and Cd36^hi^ ECs in mouse aorta

**Fig. 6:**
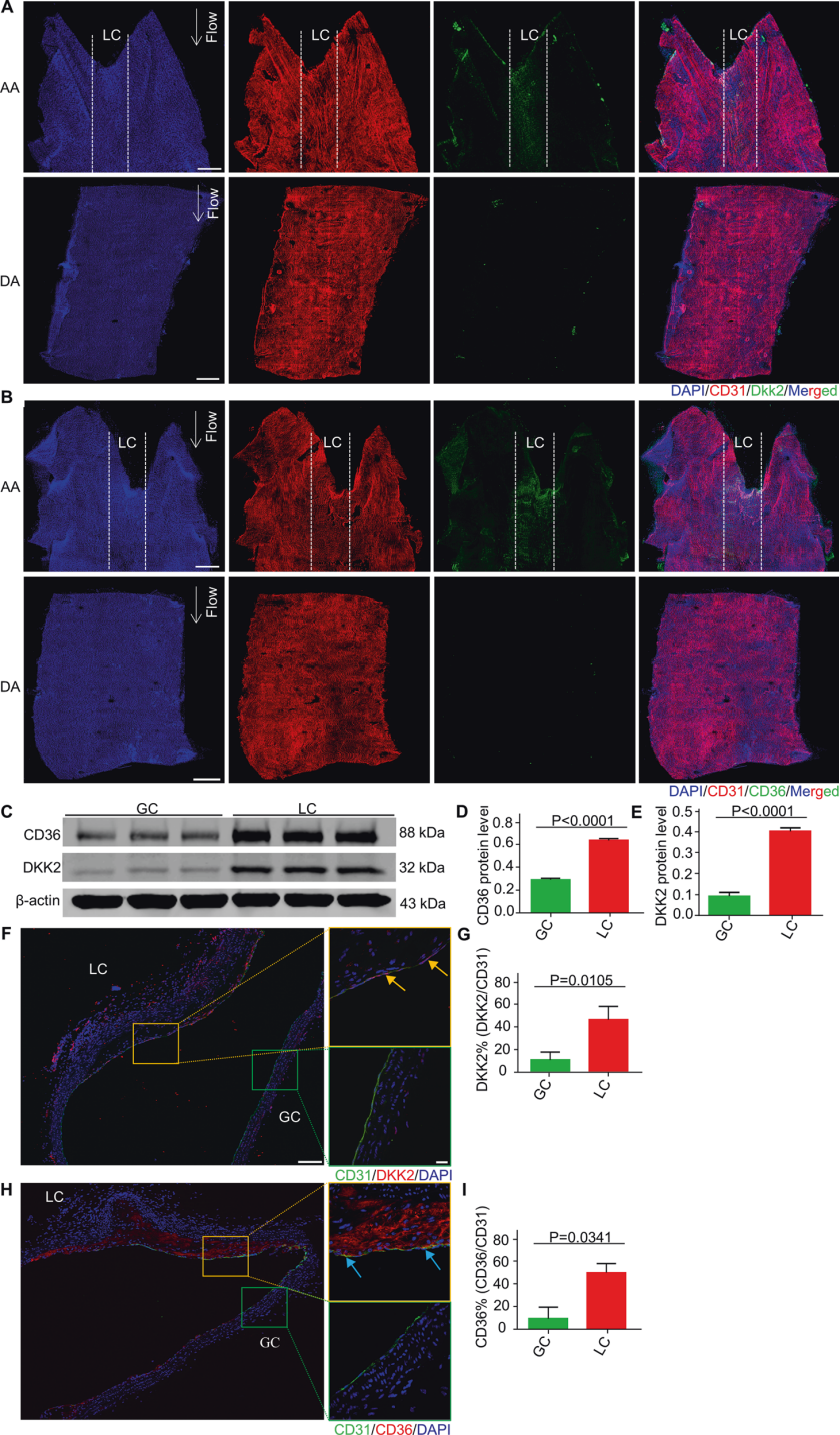
Detection of Dkk2^hi^ ECs and Cd36^hi^ ECs in mouse aorta. **A** Representative en face immunostaining images for DKK2 in the aortic arch (AA) and the descending aorta (DA) of wild-type mice (blue: DAPI; red: CD31; green: DKK2). LC indicates lesser curvatures of the aortic arch. bar = 400 µm. **B** Representative *en face* immunostaining images for CD36 in the aortic arch (AA) and the descending aorta (DA) of wild-type mice (blue: DAPI; red: CD31; green: CD36), bar = 400 µm. **C–E** The DKK2 and CD36 protein expression in the greater (GC) and lesser (LC) curvatures of wild-type mice aortic arch was analyzed by Western blotting, normalized to β-actin. Data are mean ± SD. **F-I** Representative immunofluorescence microscopy images and its statistical analysis for DKK2 or CD36 in the aortic arch of ApoE^−/−^ mice on a high-fat diet (9 weeks), bar = 100 µm. The magnified image was shown on the right, bar = 20 µm.

Although we identified Cd36^hi^ ECs and Dkk2^hi^ ECs, two novel d-flow-induced endothelial subsets, in mouse carotid artery using a partial carotid ligation (PCL) model in vivo, whether these endothelial cells present in mouse aortic arch, the proatherosclerotic area of the blood vessel where d-flow usually occurs, is unknown. We therefore examined the expression of CD36 and DKK2 in the aorta of wild-type mice. En face immunostaining showed that CD36 and DKK2 were expressed in the aortic arch, but not in the descending aorta (Fig. 6A, B). Of note, compared with the greater curvature (GC), CD36 and DKK2 were highly expressed in the lesser curvature (LC) of the aortic arch (Fig. 6A, B). Consistently, immunoblotting showed that the expression of CD36 and DKK2 protein in LC was significantly higher than GC (Fig. 6C–E).

To observe whether d-flow-induced EC subpopulations could be detected in the aorta of mice with atherosclerotic plaques, we performed immunostaining on the aortic arch from *ApoE*^*−/−*^ mice on a high-fat diet (HFD). Interestingly, we found that DKK2 and CD36 were largely co-expressed with CD31 in the LC rather than the GC (indicated by the arrow) (Fig. 6F–I). Moreover, DKK2 and CD36 were found in the atherosclerotic plaques (Fig. 6F, H). These results indicated that Cd36^hi^ ECs and Dkk2^hi^ ECs present in the region where the d-flow occurs, which may participate in atherosclerosis. Together, using the d-flow model combined with single-cell sequencing, we identified the novel subpopulations of ECs that respond to d-flow in wild-type or atherosclerotic mice.

## Discussion

By combining the PCL surgery that produces d-flow in vivo with the powerful single-cell RNA sequencing (scRNA-seq) technology, we were able to identify d-flow-induced heterogeneity of the carotid artery cells. Using Seurat-based-clustering, we uncovered 10 distinct d-flow-associated cell clusters: Dkk2^hi^ ECs, Cd36^hi^ ECs, Spp1^hi^ VSMCs, Trem2^hi^ Mφ, DCs, Birc5^hi^ Mφ, CD4^+^ T cells, CXCR6^+^ T cells, NK cells, and granulocytes. Gene expression profiles suggested Dkk2^hi^ ECs as a mechanosensitive cluster, while Birc5^hi^ Mφ a highly proliferative cluster under d-flow. Furthermore, Dkk2^hi^ ECs and Cd36^hi^ ECs present in the lesser curvature (LC) rather than the greater curvature (GC) of the aortic arch and in the atherosclerotic plaques, implying their potential role in atherosclerosis.

When exposed to d-flow, endothelial cells undergo major phenotypic changes, increased permeability, cytokine release, and leukocyte adhesion, enhancing the susceptibility to atherosclerosis [17]. Although previous studies looking into the mechanism by which endothelium responses to hemodynamic stress have unveiled an array of candidate flow responsive molecules [41], whether endothelial cells are heterogeneous in response to d-flow has not been defined. The only report in this issue was about endothelial-enriched single cells that undergo a dramatic transition from atheroprotective phenotypes to pro-inflammatory cells when d-flow occurs [42]. However, the functional significance of these heterogeneous ECs remains largely unclear. In our study, we singled out five distinct EC clusters from the carotid artery after PCL, including Klk8^hi^ ECs, Lrp1^hi^ ECs, EC 3, Dkk2^hi^ ECs, and Cd36^hi^ ECs. Klk8^hi^ ECs and Lrp1^hi^ ECs were dominantly found from the non-PCL carotid artery, whereas Dkk2^hi^ ECs and Cd36^hi^ ECs were d-flow-derived EC subpopulations. Gene expression profile of d-flow-induced Dkk2^hi^ ECs revealed that genes involved in d-flow-related biological processes, such as epithelial to mesenchymal transition [34], integrin activation [35], and transforming growth factor-beta production [36] were enriched in Dkk2^hi^ ECs, while the genes involved in lipid metabolism were enriched in Cd36^hi^ ECs, facilitating our understanding in functional significance of d-flow-induced endothelial heterogeneity.

Among the top 10 significantly enriched genes of Dkk2^hi^ ECs, Ngf, Col8a1, Kit, and Vcan were reported to be involved in the development of atherosclerosis. Whether the rest of them, including Dkk2, Ltbp2, Lamb1, Cdca7l, Dclk1, and Slc45a4, are associated with atherosclerosis and d-flow has not been reported. Importantly, we found that Dkk2 was the most upregulated gene in Dkk2^hi^ ECs induced by d-flow and highly expressed in the lesser curvature (LC) of the aortic arch under normal and pathological conditions, suggesting that DKK2 may contribute to the development of atherosclerosis by regulating endothelial function. In regard to the transition of Dkk2^hi^ ECs, single-cell trajectories analysis showed that Klk8^hi^ ECs might transform into Dkk2^hi^ ECs when ECs were exposed to d-flow. Of note, Dkk2^hi^ ECs appeared to be a mechanosensitive cluster by variation analysis of d-flow-upregulated gene set, while Klk8^hi^ ECs cluster was found in the carotid artery in response to laminar fluid shear stress, suggesting that Klk8^hi^ ECs may directly transform into Dkk2^hi^ ECs in response to d-flow. However, the underlying mechanism of Dkk2^hi^ ECs transition remains to be determined. The higher resolution of transcriptional profiling and lineage tracing may represent an attractive avenue to clarify this intriguing issue.

The significantly enriched genes (Cd36, Gpihbp1, Scarb1, and Abca1) in Cd36^hi^ ECs are involved in lipid metabolism and lipid storage. Specifically, the class B scavenger receptor Cd36 has been known to play an essential role in lipid metabolism and promote atherosclerotic lesion development [33]. Consistent with Dkk2^hi^ ECs, Cd36^hi^ ECs subpopulation was detected in d-flow-stimulated carotid artery and in the lesser curvature (LC) of the aortic arch, which is consistent with a recent report that d-flow enhances Cd36-mediated oxLDL uptake and that its expression significantly increases in the aortic arch versus descending aorta [8]. A recent scRNA-seq study surveyed all the cells in the aorta and identified two blood vessel EC subtypes, EC 1 (Vcam1^+^) and EC 2 (Cd36^+^), and a lymphatic EC cluster in the mouse aorta [26]. However, unlike our results that CD36 is highly expressed in the lesser curvature of the aortic arch, it showed a lower CD36 expression in the lesser curvature of the aortic root [26]. This discrepancy needs to be sorted out by additional or alternative experimental approaches.

Macrophage phenotypes are initially divided into proinflammatory M1 macrophages and anti-inflammatory M2 macrophages. Using scRNA-seq, we unbiasedly identified three distinct d-flow-associated macrophage subpopulations including Trem2^hi^ Mφ, Res-like Mφ, and Birc5^hi^ Mφ. Trem2^hi^ Mφ displayed enrichment of specific functions, such as lipid storage, while Res-like Mφ (M2) endowed with specialized roles in complement activation and apoptotic cell clearance in line with the previous reports [27]. Birc5^hi^ Mφ, a previously undescribed macrophage subpopulation found in the d-flow-stimulated carotid artery, highly expressed M1-associated genes (Tnf, Ccl2, Phlda1, and Cxcl10) (Figure S5B) and exhibited high proliferation capacity by maintaining the cell cycle at G2/M phase under d-flow. Among the top 10 significantly enriched genes of Birc5^hi^ Mφ, the most enriched gene was Birc5, a member of the inhibitor of apoptosis (IAP) gene family. It was reported that the Birc5 gene functions as a critical modulator of atherosclerotic macrophage apoptosis and may contribute to macrophage accumulation [43]. GO term analysis showed that cell division-related genes (Birc5, Prc1, Nusap1, Ccna2, and Ube2c) and cell proliferation-related genes (Mki67, Cdk1, Cks2, Aurkb, and Mcm10) was enriched in Birc5^hi^ Mφ. Further analysis showed that Birc5^hi^ Mφ also highly expressed genes associated with macrophage chemotaxis (Cx3cr1, Nup85, and Ccl2). The investigation on the role of Birc5^hi^ Mφ in atherosclerosis is warranted.

The vascular smooth muscle cells (VSMCs) can de-differentiate, proliferate, and migrate in response to various stimuli. Using single-cell sequencing, recent work has demonstrated that smooth muscle cells transform into fibromyocytes and macrophage-like cells in pathological conditions [29, 44]. Importantly, our scRNA-seq revealed that d-flow-stimulated Spp1^hi^ VSMCs presented a particular set of functions, such as osteoblast differentiation, blood vessel remodeling, and aging, suggesting a role in arterial stiffness. Similarly, Dkk2^hi^ ECs were found to be enriched for stiffness-associated functions, such as blood vessel remodeling. Of great interest to us is the association between endothelial stiffness promoted by d-flow [7, 8] and arterial stiffness that occurred in VSMCs that we found. Notably, endothelial microRNA-126-3p (miR-126-3p) increases SMC turnover, and its release is reduced by laminar shear stress [45], suggesting that miRs may promote arterial stiffening in VSMCs by increasing endothelial stiffness. Further study on whether the d-flow directly affects VSMCs stiffness or via EC-mediated cell communication is warranted.

Several limitations should be noticed in our study. First, due to technical constraints, we only performed bioinformatics analysis on about 2500 highly expressed genes per cell. With the increase of sequencing depth, more genes (e.g., cytokine and transcription factors) involved in the d-flow will be detected. Second, our data were from the carotid artery of mice in the early stage of d-flow (1 week). In this stage, d-flow induces endothelial dysfunction but does not lead to robust atheroma formations and advanced lesions. This allows us to detect the EC subpopulations that respond to d-flow and provide an insight into the early effects of d-flow on vascular cell heterogeneity. Thus, the PCL surgery on the carotid artery of mice with more prolonged stimulation of d-flow may generate insight toward the whole process of d-flow-induced atherosclerosis at the single-cell level. Third, enriching one type of single cells increases the depth of sequencing and facilitates typing and helps to analyze the transition of the cells as using endothelial-enriched single cells, d-flow was found to induce a dramatic transition of ECs [42]. However, the use of whole vascular tissues such as carotid artery for single-cell sequencing in this manuscript can not only obtain a vascular cell atlas but also establish the cellular communication between the different cell populations (Figure S5C). Finally, the approaches using flow-conditioned cultured cells may help understand the mechanism that is under investigation.

**Fig. 7:**
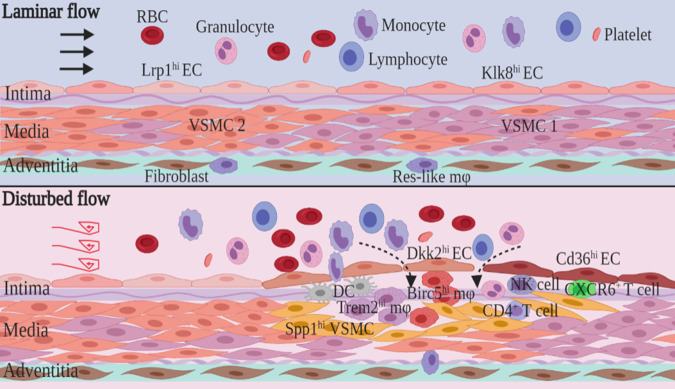
A proposed model for the cellular heterogeneity of mouse carotid artery under d-flow. In normal carotid arteries that blood flow was laminar and parallel to the blood vessel, endothelial cell subpopulations were Klk8^hi^ ECs and Lrp1^hi^ ECs, while smooth muscle cells were VSMC1 and VSMC2 clusters. When exposed to d-flow, the heterogeneity of vascular wall cells was changed compared with normal carotid arteries. New subpopulations, such as Dkk2^hi^ ECs, Cd36^hi^ ECs, and Spp1^hi^ VSMCs, were emerged. Among the infiltrating cell subpopulations, Trem2^hi^ Mφ, Birc5^hi^ Mφ, DCs, CD4^+^ T cells, CXCR6^+^ T cells, NK cells, and granulocytes were identified under d-flow.

In conclusion, we established the transcriptional landscape of d-flow-induced cell subpopulations in the mouse carotid artery and identified 10 distinct clusters related to d-flow: Dkk2^hi^ ECs, Cd36^hi^ ECs, Spp1^hi^ VSMCs, and infiltrating cell subpopulations including Trem2^hi^ Mφ, DCs, Birc5^hi^ Mφ, CD4^+^ T cells, CXCR6^+^ T cells, NK cells, and granulocytes (Fig. 7). Gene set variation analysis of these subpopulations revealed specialized functions, such as integrin activation, osteoblast differentiation, leukocyte chemotaxis, complement activation, immune response, leukocyte aggregation, and cell killing among different clusters. Dkk2^hi^ ECs and Cd36^hi^ ECs were also detected in the mouse aorta under normal and pathological conditions. Targeting d-flow-induced cell subpopulations and their specialized functions may provide potential new therapeutic directions for atherosclerosis.

## Materials and Methods

### Reagents and Mice

Collagenase Type II was purchased from Sigma-Aldrich (Sigma-Aldrich, Cat#C6885-100MG). Deoxyribonuclease I was purchased from Worthington (Worthington, Cat#LS002139). Trypsin was purchased from Beyotime (Beyotime, Cat#C0205). Other reagents used in this study include Hank’s Balanced Salt Solution (Thermo Fisher Scientific, Cat#14025092), PBS (Solarbio, Cat#P1000), Heparin sodium salt (Solarbio, Cat#H8060), Fetal bovine serum (HyClone, Cat#SV30087.03).

All mouse studies were approved by the Institutional Animal Care and Use Committee of Soochow University, and all protocols complied with institutional guidelines. *ApoE*^*-/-*^ and wild-type mice (C57BL/6 J background) from the Jackson Laboratories (Bar Harbor, USA) were kept as previously described [2].

### Partial carotid artery ligation

We performed partial carotid artery ligation (PCL) surgery as previously described [17]. Briefly, the external carotid artery (ECA), internal carotid artery (ICA), and occipital artery (OA) of the left carotid artery (LCA) of 8-week-old mice were ligated with 10–0 silk suture while the superior thyroid artery (STA) of LCA was left untouched, and the ultrasound was used to monitor the flow reversal in LCA. Seven days after PCL, the left carotid arteries with or without PCL were harvested.

### Cell suspension preparation

Mice were euthanized by CO_2_ and blood was drawn. The remaining blood in the LCA was perfused from the left ventricle with 50 mL PBS containing 2.5 U/mL heparin sodium. Using a dissecting microscope, the isolated LCA was carefully chopped and washed twice in 2 mL ice-cold PBS. The diced vascular tissue was collected and incubated in a solution containing dissociation enzyme (1 mg/mL collagenase type II, 0.02 mg/mL deoxyribonuclease I) for 1 h at 37 °C. Then, 500 µL 1.5% FBS-PBS was added and the cell suspension was filtered through a 40 µm sterile cell strainer (JETBIOFIL, Cat#css010040). Cells were spun down at 1000 rpm for 5 min at 4 °C. To prepare a single-cell suspension, 0.12% trypsin solution was used to resuspend cells and incubated for another 5 min at 37 °C. The cells were resuspended in 200 µL ice-cold PBS. Then, cell concentration and viability were evaluated by AOPI Dual-fluoresces counting (Figure S1).

### Single-cell RNA sequencing

Using Single Cell 3’ Library, Gel Bead Kit V3 (10x Genomics, 1000075), and Chromium Single Cell B Chip Kit (10x Genomics, 1000074), single cells (500–1000 live cells per microliter determined by Count Star) were loaded on a Chromium Single Cell Controller (10x Genomics) to generate single-cell gel beads in emulsion (GEMs) following the manufacturer’s protocol. Briefly, single cells were resuspended in PBS with 0.04% BSA and added to each channel. The captured cells were lysed, and the released RNA was barcoded through reverse transcription in individual GEMs. Barcoded cDNA was amplified, and the quality was controlled using Agilent 4200 TapeStation System. scRNA-seq libraries were prepared using Single Cell 3′ Library and Gel Bead Kit V3 following the manufacture’s introduction. Sequencing was performed on an Illumina Novaseq 6000 sequencer with a pair-end 150 bp (PE150) reading strategy (performed by CapitalBio Technology, Beijing).

### Single-cell data preprocessing

Alignment, filtering, barcode counting, and UMI counting were performed with Cell Ranger to generate a feature-barcode matrix and their global gene expressions. Dimensionality reduction, visualization, and analysis of scRNA-sequencing data were performed with the R package Seurat (version 3.1.2). Cells whose expression of <200 or >4000 genes or mitochondrial gene ratio that was more than 10% were regarded as abnormal and filtered out. Two thousand highly variable genes were used for downstream clustering analysis. Principal Component Analysis (PCA) was performed, and the number of the significant principal components was calculated using the built-in “ElbowPlot” function. Cell types were identified by using CellMarker. Data visualization in two dimensions were realized by t-SNE.

### Single-cell trajectories

Single-cell trajectories were analyzed by the Monocle package to discover developmental transitions. We used highly variable genes identified by Seurat to sort cells into pseudotime order. Using “orderCells” functions, we recognized the state of cells at the start point of the pseudotime and set this state as the root_state argument. “DDRTree” was applied to reduce dimensions. Differentially expressed genes over the pseudotime were calculated by the “differentialGeneTest” (*P* value <10^−^^20^) and visualized by “plot_cell_trajectory”.

### Cell-cycle analysis

The cell-cycle phase was assessed by “CellCycleScoring” in Seurat. G1/S and G2/M states of each cell were defined by comparing the average expression of the cell-cycle-related gene sets.

### En face immunostaining

The aorta and common carotid arteries were fixed in 4% paraformaldehyde (PFA) for 2 h and washed with 0.3% PBSTX (0.3% Triton X-100 in PBS, V/V) for four times, 15 min each at room temperature (RT). Tissues were then blocked with 3% PBSMT (3% milk in 0.3% PBSTX) at 4 °C overnight and blotted with primary antibody (diluted in 0.3% PBSTX) in blocking buffer at 4 °C overnight. After washed with 0.3% PBSTX for four times, tissues were incubated with fluorescent-conjugated secondary antibody (diluted in 0.3% PBSTX) at 4 °C overnight. After washed with 0.3% PBSTX for four times, tissues were fixed with 1% PFA for 3 min and washed with 0.3% PBSTX for four times. Finally, tissues were mounted with cover slides and sealed with an antifading agent. The primary antibodies used in the en face immunostaining, include rat antimouse CD31 (1 µg/mL, BD Biosciences, Cat#553370), rabbit antimouse DKK2 (1:50, Novus Biologicals, Cat#NBP2-68703), and mouse antimouse CD36 (5 µg/mL, Abcam, Cat#ab23680).

### Immunofluorescence

The carotid arteries were fixed in 4% PFA overnight and washed with PBS for three times, 5 min each before dehydrating with 20% sucrose. On the next day, tissues were embedded in optimal cutting temperature (OCT) compound and stored at −80 °C. The frozen samples were sectioned into 10-µm-thick horizontal slices using a Leica CM1950 cryosectioning instrument (Leica Microsystems). The sections were incubated with primary antibody (mouse antimouse CD68 (1:100, Abcam, Cat#ab955), rabbit antimouse BIRC5 (1:100, Abcam, Cat#ab134170), rabbit antimouse TREM2 (4 µg/ml, Proteintech, Cat#13483-1-AP), α-smooth muscle-Cy3™ antibody (2 µg/mL, Sigma-Aldrich, Cat#C6198), mouse antimouse TSP-1 (2 µg/mL, Santa Cruz, Cat#sc-59887), mouse antimouse CTGF (1 µg/mL, Santa Cruz, Cat#sc-101586), or an IgG control at 4 °C overnight for immunofluorescence staining. The fluorescent secondary antibodies (goat antimouse IgM mu chain-Alexa Fluor® 647, abcam, Cat#ab150123, or donkey antirabbit IgG H&L-Alexa Fluor® 568, abcam, Cat#ab175470) and DAPI (SouthernBiotech, Cat#0100-01) were used to visualize specific proteins. Images were filmed using a multicolor digital camera on the FV3000 confocal microscope (Olympus, Japan).

### Immunoblotting

The lesser (LC) or greater (GC) curvature regions of the mouse aortic arch was lysed in RIPA buffer (1% Triton X-100, 1% deoxycholate, 0.1% SDS, 10 mM Tris and 150 mM NaCl) with protease and phosphatase inhibitor cocktail (Thermo Fisher, Cat#78440). Proteins samples (40 µg) were heated at 95 °C for 5 min in sample buffer (161-0737, Bio-Rad, USA) and separated in 10% SDS-PAGE gels. Western blots were incubated with 5% nonfat milk (Solarbio, Cat#D8340), washed with TBST, and probed with primary antibodies: rabbit antimouse DKK2 (1:500, Proteintech, Cat#21051-1-AP), goat antimouse CD36 (0.1 µg/mL, R&D Systems, Cat#AF2519), or rabbit antimouse β-actin (1:20000, ABclonal, Cat#AC026). After incubation at 4 °C overnight, membranes were incubated with secondary antibodies: donkey antigoat IRDye 800CW (1:10000, LI-COR Biosciences, Cat#925-32214) or goat antirabbit IgG (H + L) (DyLight™ 800 4X PEG Conjugate, 1:10000, Cell Signaling Technology, Cat#5151 S). Proteins were detected using the Odyssey infrared imaging system (LI-COR Biosciences, USA).

### Data analysis

Marker genes in scRNA-seq profiles with a minimum log-fold change threshold of 0.25 and with *P* values computed with a Wilcoxon rank-sum test were calculated by the “FindAllMarkers” function in the Seurat package. Using Molecular Signatures Database (MSigDB), we applied GSVA to score biological processes and canonical pathways between different clusters. Besides, we also used DAVID to perform biological process enrichment analysis with the top 100 highly expressed marker genes. The results were visualized using the R package, including ggplot2 and pheatmap. Image J software (NIH) was used to quantify protein expression levels with β-actin as internal control and analyzed by Prism 8.0 GraphPad software.

## Supplementary information

Supplemental figure legends

Figure S1

Figure S2

Figure S3

Figure S4

Figure S5

## Data Availability

Single-cell RNA sequencing data that support the findings of this study have been deposited in SRA with the accession code PRJNA722117.
