# Supplementary material for: Single-cell RNA-seq reveals cellular heterogeneity of mouse carotid artery under disturbed flow
Source: Cell Death Discov. 2021 Jul 16;7:180. doi: 10.1038/s41420-021-00567-0 (PMC8290019; doi:10.1038/s41420-021-00567-0)
Supplement: Supplementary file 1 — Supplemental figure legends [file 41420_2021_567_MOESM1_ESM.docx]

**Supplementary Figure Legends**

**Figure S1: A)** The cell viability of the two groups after enzyme digestion. AOPI dual-fluoresence counting was used for detecting cell concentration and viability. The results showed that nucleated cells stained with fluorescent green are counted as alive, whereas nucleated cells stained with fluorescent red are counted as dead by the Countstar® Rigel software. **B)** The cell size of the three groups was measured after enzyme digestion. AO, Acridine orange; PI, Propidium iodide.

**Figure S2: A)** Overview of single-cell RNA-sequencing data quality metrics from left carotid artery of wild type mice with or without partial carotid ligation (PCL). **B)** Plots of the number of UMIs per cell (nCount_RNA) and the proportion of mitochondrial genes (percent.mt) in PCL and control groups.

**Figure S3: A)** Stacked bar chart showing the relative abundance of the 6 major cell types identified by scRNA-seq of PCL and control groups. **B)** T-Distributed Stochastic Neighbor Embedding (t-SNE) plots of the marker genes expression in various clusters from left carotid artery of wild type mice with partial carotid ligation (PCL) and control.

**Figure S4: A)** Violin plots showing the Klk8 and Lrp1 expression in all 5 EC clusters. **B)** Representative en face immunostaining images for CD36 and DKK2 in left carotid artery with partial carotid ligation (left) or without PCL (control) (right) (red: CD31; blue: CD36; green: DKK2). bar= 400 μm. **C)** Violin plots of Lmo4, Angpt2, Icam1, Ctgf, Ctps, Sema7a, Fosl2 and Bmp4 expression in all 5 EC clusters. **D)** Violin plots of Icam2, Dhh, Klf2, Klf4, Klk10, Nos3, Kras and Ptprj expression in all 5 EC clusters.

**Figure S5: A)** Gene ontology enrichment analysis of biological processes in Mφ/DCs (Top 5). **B)** Violin plots of Tnf, Ccl2, Phlda1 and Cxcl10 expression in 4 distinct Mφ/DCs populations. **C)** Putative ligand and receptor-based cell-cell interaction among the carotid artery cells under d-flow (performed by CellPhoneDB). In general, most of the subpopulations formed interconnected networks. The VSMC versus Fibro or EC as well as EC versus Fibro showed strong correlations.
