# Supplementary figures and images for: Single-cell RNA-seq reveals cellular heterogeneity of mouse carotid artery under disturbed flow
Source: Cell Death Discov. 2021 Jul 16;7:180. doi: 10.1038/s41420-021-00567-0 (PMC8290019; doi:10.1038/s41420-021-00567-0)

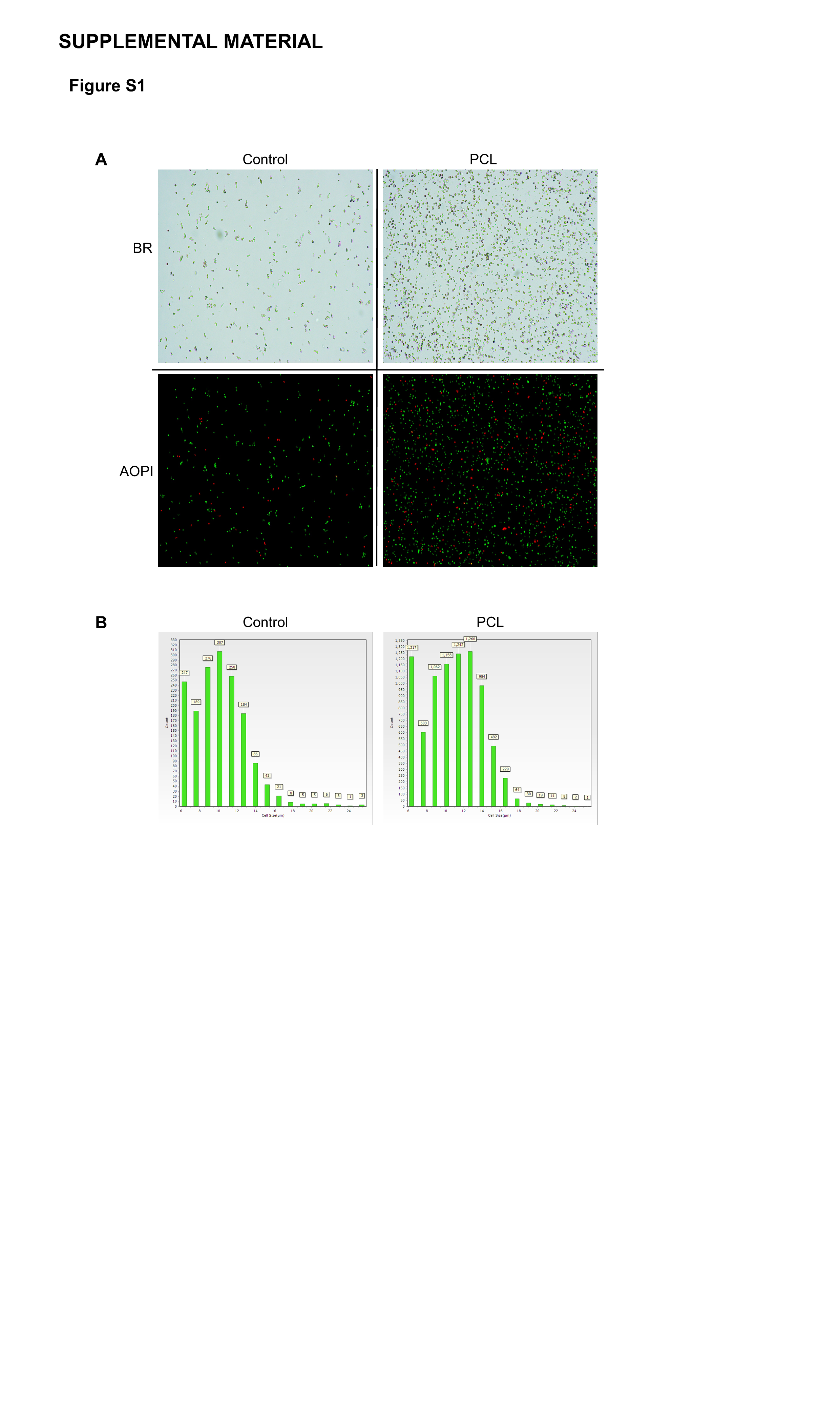

Supplement: Supplementary file 2 — Figure S1 [file 41420_2021_567_MOESM2_ESM.tif]

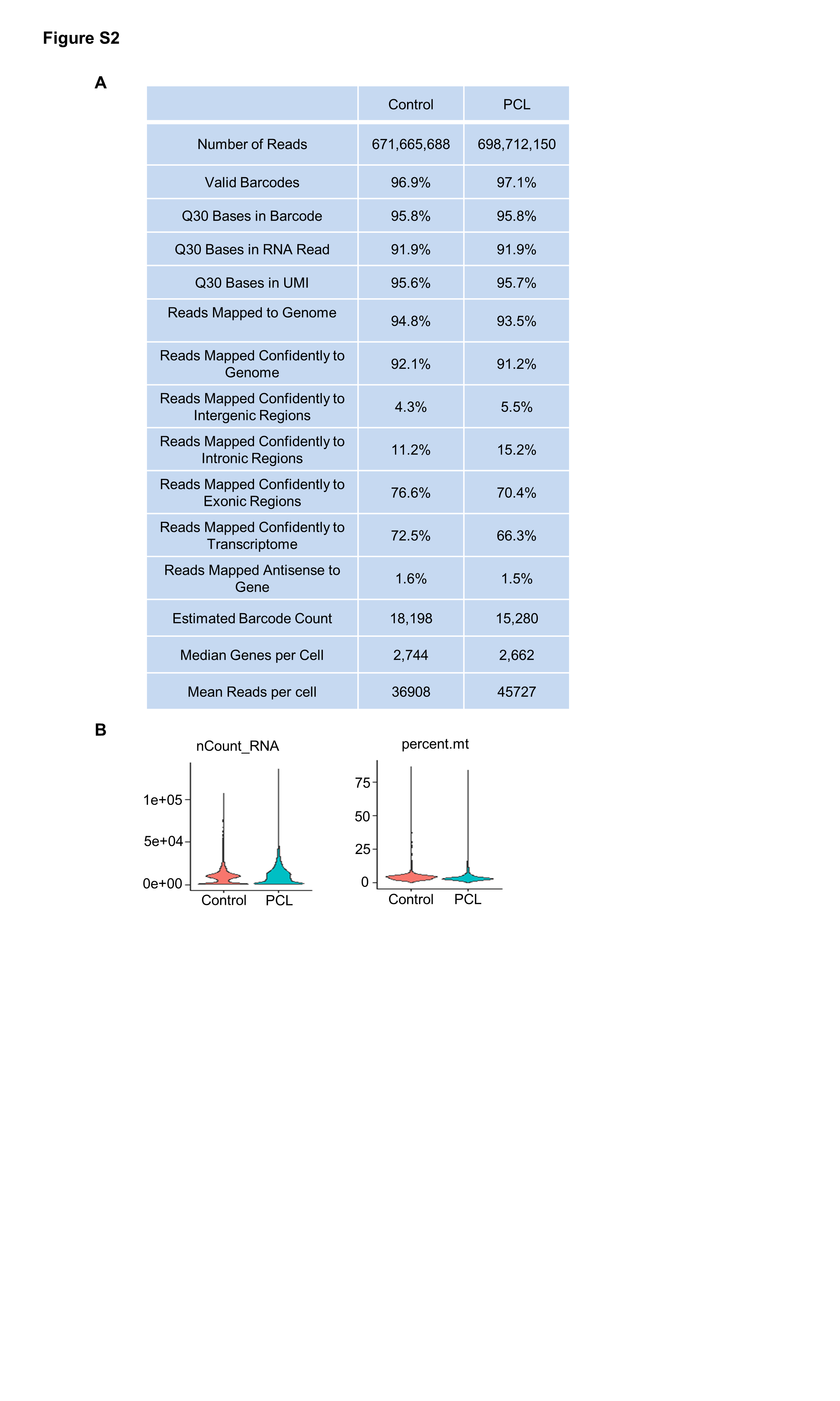

Supplement: Supplementary file 3 — Figure S2 [file 41420_2021_567_MOESM3_ESM.tif]

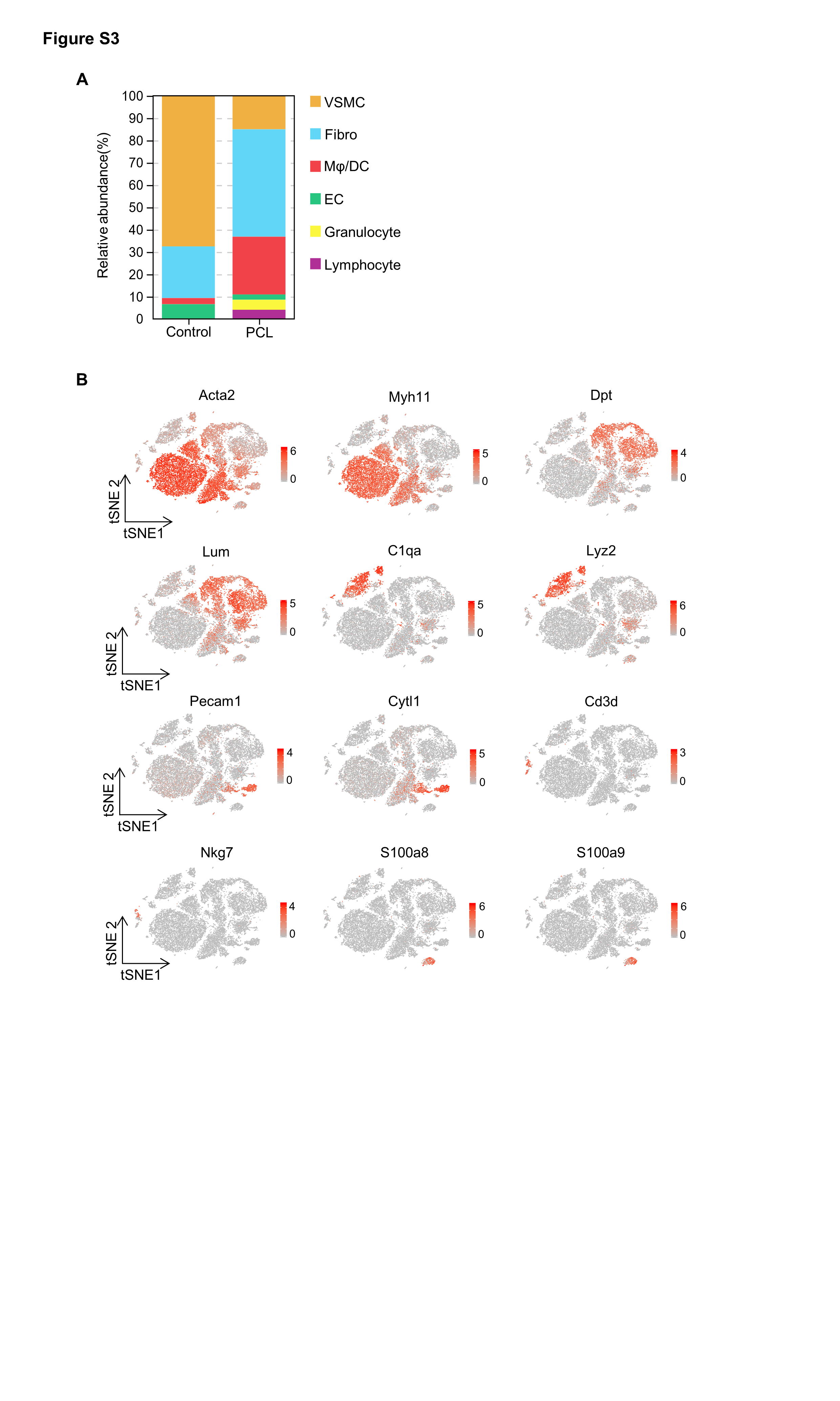

Supplement: Supplementary file 4 — Figure S3 [file 41420_2021_567_MOESM4_ESM.tif]

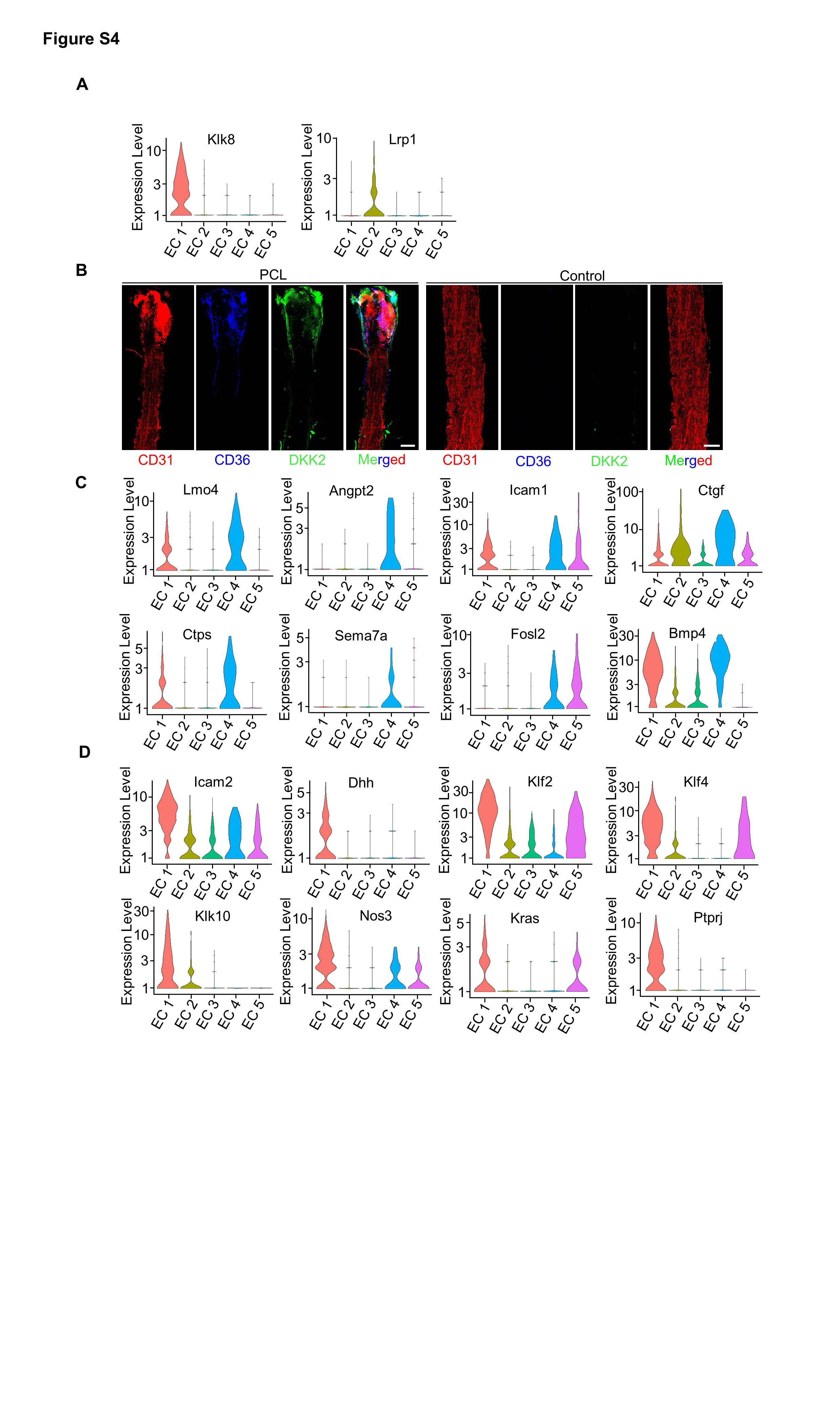

Supplement: Supplementary file 5 — Figure S4 [file 41420_2021_567_MOESM5_ESM.tif]

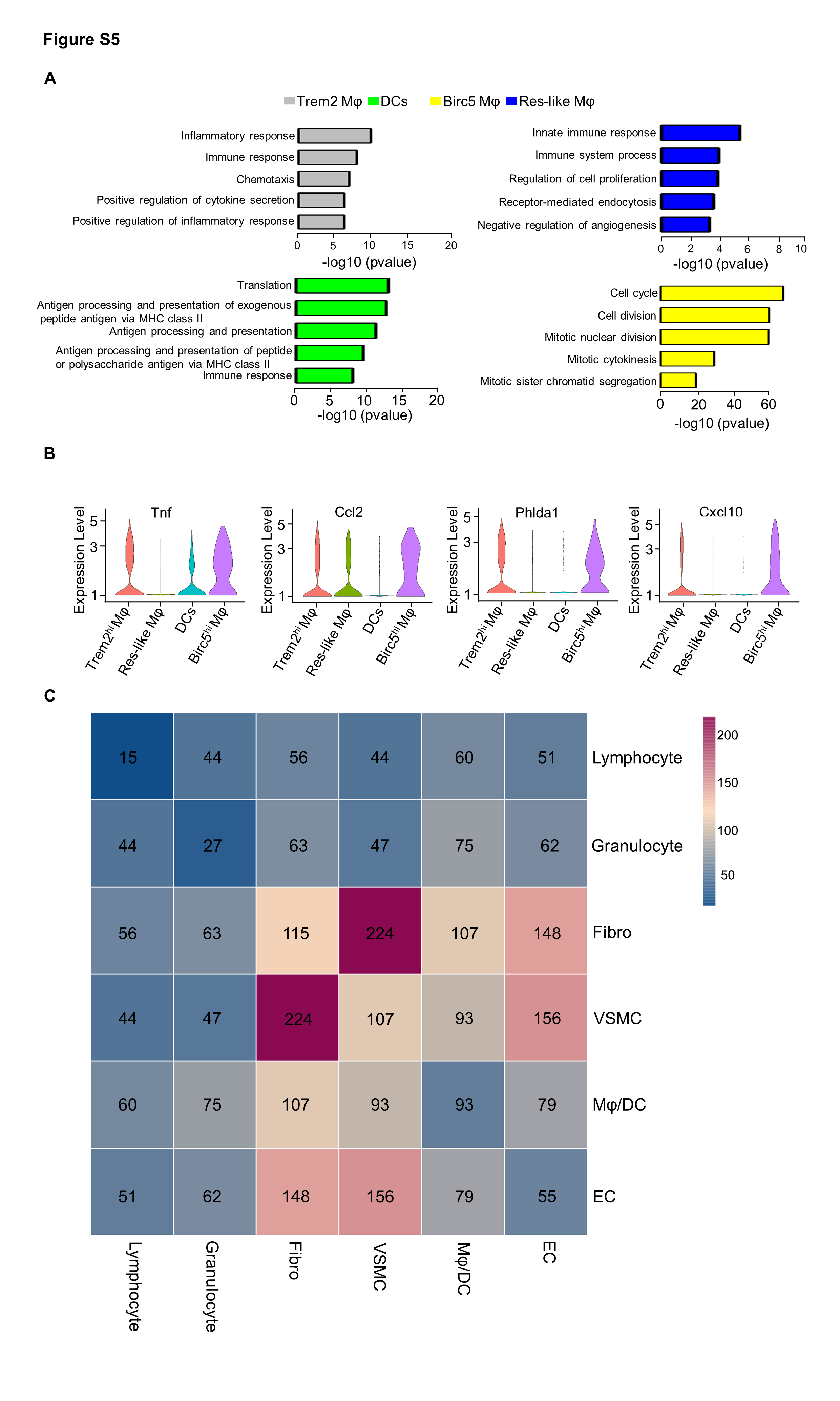

Supplement: Supplementary file 6 — Figure S5 [file 41420_2021_567_MOESM6_ESM.tif]
